# Multimethod investigation of the neurobiological basis of ADHD symptomatology in children aged 9-10: baseline data from the ABCD study

**DOI:** 10.1038/s41398-020-01192-8

**Published:** 2021-01-18

**Authors:** Max M. Owens, Nicholas Allgaier, Sage Hahn, DeKang Yuan, Matthew Albaugh, Shana Adise, Bader Chaarani, Joseph Ortigara, Anthony Juliano, Alexandra Potter, Hugh Garavan

**Affiliations:** Department of Psychiatry, University of Vermont, Burlington, VT 05401 USA

**Keywords:** Predictive markers, ADHD

## Abstract

Attention deficit/hyperactivity disorder is associated with numerous neurocognitive deficits, including poor working memory and difficulty inhibiting undesirable behaviors that cause academic and behavioral problems in children. Prior work has attempted to determine how these differences are instantiated in the structure and function of the brain, but much of that work has been done in small samples, focused on older adolescents or adults, and used statistical approaches that were not robust to model overfitting. The current study used cross-validated elastic net regression to predict a continuous measure of ADHD symptomatology using brain morphometry and activation during tasks of working memory, inhibitory control, and reward processing, with separate models for each MRI measure. The best model using activation during the working memory task to predict ADHD symptomatology had an out-of-sample *R*^2^ = 2% and was robust to residualizing the effects of age, sex, race, parental income and education, handedness, pubertal status, and internalizing symptoms from ADHD symptomatology. This model used reduced activation in task positive regions and reduced deactivation in task negative regions to predict ADHD symptomatology. The best model with morphometry alone predicted ADHD symptomatology with an *R*^2^ = 1% but this effect dissipated when including covariates. The inhibitory control and reward tasks did not yield generalizable models. In summary, these analyses show, with a large and well-characterized sample, that the brain correlates of ADHD symptomatology are modest in effect size and captured best by brain morphometry and activation during a working memory task.

## Introduction

There is substantial evidence that individuals with Attention Deficit Hyperactivity Disorder (ADHD) have below average working memory^1–3^ and difficulty with response inhibition^4–6^. These deficits may be explained by reduced activation in brain regions, such as the dorsolateral prefrontal cortex (DLPFC), anterior cingulate cortex (ACC), posterior parietal cortex (PPC), ventrolateral prefrontal cortex (VLPFC), insula thalamus, and striatum, which have been found to differ in individuals with ADHD in meta-analyses of functional magnetic resonance imaging (fMRI) tasks of inhibitory control, working memory, and attention^7–9^. Differences in individuals with ADHD have also been found in activation in the ventral striatum, ventromedial prefrontal cortex (VMPFC), and ACC during monetary reward tasks^10^. Additionally, a recent review of morphometric differences measured by structural magnetic resonance imaging (sMRI) in adolescents with ADHD reported abnormalities in cortical thickness (CT) in the lateral prefrontal and parietal cortices, as well as gray matter volume of the thalamus as the most frequently identified effects^11^. However, these reviews and meta-analyses included few studies with more than 50 participants and none over 100.

Two recent large studies of adolescents (*N* > 1000) have aimed to characterize the neuroanatomical correlates of ADHD. These studies both investigated surface-based morphometry in a non-clinical sample of adolescents using the IMAGEN study dataset (https://imagen-europe.com/), which includes participants with a range of ADHD symptoms. They identified lower levels of CT in the ventromedial prefrontal cortex^12,13^ and cortical surface area (CSA) in the DLPFC and ACC^14^, which were also linked to performance on several neuropsychological measures known to be disrupted in individuals with ADHD. Additionally, a recent mega-analysis (*N* = 1713 with ADHD, 1529 controls) found smaller gray matter volume (GMV) in children with ADHD in several subcortical structures (nucleus accumbens, caudate, putamen, amygdala, and hippocampus)^15^. However, comparably powered studies have not been published for task fMRI.

In response to concerns of mounting replication failures in the social sciences, reducing overfitting and assessing generalizability of models have become critical. To achieve this, methods such as regularized regression and cross-validation are frequently used^16^. A recent large machine learning study (*N* = 2713) found only modest prediction accuracies when distinguishing children with ADHD from controls using sMRI with more rigorous cross-validation^17^. It stands in contrast to prior smaller studies, which had shown much more optimistic predictions using more questionable approaches (e.g., failure to tune regularization hyperparameters, not validating tuned hyperparameters on an independent sample, and using accuracy as an outcome for imbalanced groups). However, the majority of existing machine learning work on ADHD focuses on only one imaging modality or task, making it difficult to compare classification accuracies across ADHD studies. Additionally, the use of non-linear modeling strategies makes the interpretation of model features in many machine learning models difficult.

Another approach to improving replicability has been the initiation of large-scale open-data projects. The current study used baseline data from the Adolescent Brain and Cognitive Development (ABCD; www.ABCDstudy.org) study to predict current ADHD symptomatology using elastic net regression models with brain activation during fMRI tasks of working memory, inhibitory control, and the anticipation and receipt of monetary reward, as well as brain morphometry measured by sMRI. Among machine learning approaches, elastic net regression is particularly effective at making predictions when the predictors are highly intercorrelated, has been shown to be effective at making predictions using neuroimaging data^18^, and is one of the approaches in which model features are most interpretable, making it ideal for attempting to understand the specific brain regions most critical to ADHD. The current analyses also used cross-validation with an external test set to ensure that effects identified were replicable in an independent dataset.

An additional weakness in the existing literature on the neurobiological basis of ADHD is a primary focus on categorical diagnoses of ADHD. There is strong empirical evidence that child psychopathology is better suited to dimensional measurement^19^. This is true specifically for ADHD as well^20^. Sub-diagnostic ADHD symptomatology has been linked to poorer educational outcomes^21^ and differences in cortical maturation in children^22,23^. Continuous measures of ADHD symptomatology show stronger genetic correlations than ADHD diagnoses^24^ suggesting that continuous measures are investigating the same construct at a higher resolution (as opposed to another phenomenon altogether). Furthermore, the use of a dimensional approach is more consistent with the National Institute of Mental Health’s Research Domain Criteria program^25^. Thus, the current study used a continuous measure of ADHD symptomatology, the “attention problems” scale of the child behavior checklist (CBCL)^26^.

Given the established relationship of working memory, inhibitory control, and reward processing with ADHD, we hypothesized that ADHD symptomatology would be predictable using each of the three fMRI tasks. We expected that the features used by these models would be regions activated by the tasks in prior studies, such as the DLPFC, VLPFC, and ACC for working memory and inhibition tasks and the striatum, VMPFC, and ACC for the reward task. Based on prior work linking structural MRI data to ADHD, we also hypothesized that structural MRI would predict ADHD symptomatology. We expected that the features used in these models would be the same as those whose morphometry had been found to be associated with ADHD in prior studies, such as the DLPFC, ACC, VMPFC, and striatum.

## Materials and methods

### Procedures

The Adolescent Brain Cognitive Development^sm^ Study (ABCD) is an ongoing multisite, longitudinal neuroimaging study following a cohort of 11,875 youths over 10 years. In this analysis, we use data from the baseline visits at which participants were 9–10 years old. All data used in the current study (i.e., fMRI, questionnaire, neuropsychological tasks) were collected at a single visit or across two visits that occurred within 30 days of each other. The ABCD study® was approved by the institutional review board of the University of California, San Diego (IRB# 160091). Additionally, the institutional review boards of each of the 21 data collection sites approved the study. Informed consent was obtained from all parents and informed assent was obtained from participants. Data can be accessed through registration with the ABCD study at https://nda.nih.gov/abcd.

### Participants

Demographics for the full sample are shown in Table 1 and the distribution of ADHD symptomatology is shown in Supplemental Fig. 1. While the ABCD Data Analysis, Informatics & Resource Center (DAIRC) creates several indices of data quality, all exclusions were done by the research team of the current study starting from the total 11,875 participants enrolled in the ABCD study. Participants for all MRI/fMRI modalities were excluded if they had incomplete data for the CBCL “Attention Problems” scale, were missing sociodemographic covariates, or failed the FreeSurfer quality control assessments performed by the DAIRC. In the DAIRC Freesurfer quality control, trained technicians reviewed each subject’s cortical reconstruction, judging the severity of five problem types: motion, intensity inhomogeneity, white matter underestimation, pial overestimation, and magnetic susceptibility artifact. Reconstructions were rated for each problem as “none”, “mild”, “moderate”, or “severe”. A subject was recommended for exclusion if any of the five categories are rated as “severe” and these recommendations were summarized as an overall binary QC score (i.e., pass or fail), which was used in the current manuscript^27^. For each fMRI task, participants were excluded for having any missing fMRI data on that task, having fewer than two fMRI scans pass the image quality control performed by the DAIRC (which was similar to the DAIRC Freesurfer QC reported above^27^), or failing to meet additional quality control criterion specific to this report. These additional quality control steps included having: (1) hemispheric mean beta-weights more than two standard deviations from the sample mean, (2) fewer than 200 degrees of freedom over the two runs, (3) mean framewise displacement > 0.9 mm for both runs, (4) failed to meet task-specific performance criteria (described in Casey et al.^28^). Because of a data processing error (https://github.com/ABCD-STUDY/fMRI-cleanup), participants were excluded who were collected on Philips scanners for all fMRI tasks. Additionally, for the SST only, a small group of participants were excluded because of a glitch in the SST task (when the stop signal delay is 50 msec, a response that is faster than 50 msec is erroneously recorded as the response for all subsequent Stop trials, see Garavan et al.^29^). This resulted in participant totals of 8596 for Structural MRI (sMRI), 5417 for the EN-Back task, 5959 for the monetary incentive delay (MID) task, and 5020 for Stop Signal Task (SST). Participant exclusion steps are reported in Supplemental Table 1.

**Table 1:** Descriptive statistics on samples for each MRI paradigm.

	sMRI		EN-Back		SST		MID	
	Mean	SD	Mean	SD	Mean	SD	Mean	SD
Child age								
Months	119.18	7.46	119.61	7.53	119.53	7.54	119.42	7.52

	Total	%	Total	%	Total	%	Total	%
Child sex								
Female	4179	49%	2681	49%	2524	50%	2973	50%
Child race								
White	5917	69%	3878	72%	3596	72%	4174	70%
Black	1140	13%	592	11%	570	11%	712	12%
Asian	177	2%	111	2%	105	2%	118	2%
Other	1362	16%	836	15%	749	15%	955	16%
Child ethnicity								
Hispanic	1604	19%	997	18%	911	18%	1122	19%
Household income								
<$50 K	2303	27%	1313	24%	1228	24%	1514	25%
$50K–$100 K	2474	29%	1588	29%	1462	29%	1736	29%
>$100 K	3819	44%	2516	46%	2330	46%	2709	45%
Parent education								
<HS diploma	283	3%	142	3%	136	3%	169	3%
HS diploma/GED	644	7%	352	6%	338	7%	421	7%
Some college	2153	25%	1303	24%	1197	24%	1465	25%
Bachelor	2336	27%	1547	29%	1434	29%	1675	28%
Post graduate degree	3180	37%	2073	38%	1915	38%	2229	37%

### Measures

#### Child behavior checklist

The “Attention Problems” empirically derived syndrome scale of the CBCL parent-report questionnaire was used to evaluate symptoms of ADHD symptomatology as a continuous variable^30^. Despite its name, the CBCL “Attention Problems” scale evaluates a broad constellation of ADHD symptomatology (attention, hyperactivity, and impulsivity) and has been shown to be an excellent predictor of ADHD diagnosis derived from clinical interview^31,32^. Additionally, the CBCL internalizing composite was used as a covariate to account for depression and anxiety, which are frequently comorbid in children with ADHD. This composite is made up of the anxious/depressed scale, the withdrawn/depressed scale, and the somatic symptoms scale.

#### Demographic Questionnaire

A demographics questionnaire was administered to the participant’s parent/guardian to determine demographic information including sex, age, race, household income, and parental education.

#### Pubertal Development Scale

Pubertal status was assessed using the pubertal development scale^33^, which was completed by a parent/guardian and by the participant, with results of the two being averaged. This measure has been shown to have good reliability and to correspond with accepted self-report and biological measures of pubertal development^33^.

#### Edinburgh handedness inventory - short form

The Edinburgh Handedness Inventory – Short Form^34^ is a 4-item self-report scale that produces a handedness score of “right”, “mixed”, or “left” by asking about preferred hand used for writing, throwing, using a spoon, and using a toothbrush. The measure has been shown to have good reliability and to correlate highly with longer measures of handedness^34^.

#### Medications survey inventory

Parents completed a survey in which they listed the names and dosages of all medications taken by the child. From this we created a binary variable if participants were taking one or more stimulant medications prescribed to treat ADHD (e.g., Adderall, Ritalin, amphetamine). For a full list of medications included in this variable, see Supplemental Table 2.

#### fMRI tasks

The tasks used in the current study have been described previously^28,35^ and are detailed in Supplemental Materials and a schematic of the tasks is shown in Supplemental Fig. 2. In short, the EN-Back task was a modified version of a traditional N-Back task in which participants viewed a series of stimuli and for each responded if that stimulus matched the one they saw *N* items ago (i.e., “*N* back”). The current task version incorporated added elements of facial and emotional processing, though these were not a focus of the current analysis. The task had two conditions: a 2-back as its active condition and a 0-back as the baseline condition. d’ (*z*(hit rate) − *z*(false alarm rate)) was used as the performance measure for the EN-back tasks. The MID task included both anticipation and receipt of reward and loss. In this task, participants viewed an incentive cue for 2 sec (anticipation) and then quickly respond to a target to win or avoid losing money ($5.00 or $0.20). Participants were then given feedback about their performance (receipt). The baseline used was “neutral” trials in which participants completed the same action but with no money available to be won or lost. The current study focused only on reward trials (i.e., trials in which participants win money), as most prior research on ADHD symptomatology has focused on reward trials^10^. The SST consisted of serial presentations of leftward and rightward facing arrows. Participants were instructed to indicate the direction of the arrows using a two-button response box (the “go” signal), except when the left or right arrow was followed by an arrow pointing upward (the “stop” signal). Participants were also instructed to respond as “quickly and accurately as possible”. Trials were then categorized based on the participant’s accuracy (“correct” and “incorrect”). The performance variable used for the SST was stop signal reaction time (SSRT), which represents the duration required to inhibit a “go” response after a “stop signal” has been presented and functions as an index for inhibitory speed.

#### Magnetic resonance imaging acquisition and data processing

Structural and functional MRI scans were acquired at sites across the United States using 26 different scanners from two vendors (Siemens and General Electric); there were also three sites using Philips scanners that were excluded from analyses due to an error in processing prior to their release. MRI sequences are reported in Supplemental Methods and in prior work^31^. All sMRI and fMRI data were preprocessed by the DAIRC using pipelines that have been detailed in prior work^27^. Briefly, sMRI data were preprocessed using FreeSurfer version 5.3^27^ to produce CT and CSA measures for each of the 74 Destrieux atlas^36^ regions of interest in each hemisphere (148 regions total) and GMV for nine subcortical regions in FreeSurfer’s ASEG parcellation in each hemisphere (18 regions total), plus the brainstem which was not split by hemisphere. All structural MRI data were visually examined by a trained ABCD technician, who rated them from zero to three on five dimensions: motion, intensity homogeneity, white matter underestimation, pial overestimation, and magnetic susceptibility artifact. From this an overall score was generated recommending inclusion or exclusion^27^. All subjects recommended for exclusion based on their Freesurfer data were excluded from all analyses in the current study, including fMRI analyses as the fMRI processing pipeline relies on the Freesurfer cortical reconstruction.

fMRI data were preprocessed using a multi-program pipeline that yielded neural activation in these same cortical and subcortical regions for each fMRI contrast. The contrasts used for the SST were *incorrect stop – correct go* and *correct stop – correct go*; for the EN-Back the only contrast was *2-Back vs. 0-Back*; for the MID, contrasts were *reward anticipation – neutral anticipation* and *positive reward outcome – negative reward outcome* (i.e., *win* – *loss)*.

### Data analysis

#### Preliminary analyses

To investigate the relationship between covariates and ADHD symptomatology, we examined the association of each covariate with ADHD symptomatology in a separate mixed effect model for each covariate using R version 3.6.1. Code for all analyses is available at https://github.com/owensmax/ADHD. Covariates were participants’ age, sex, race, pubertal status, handedness, internalizing symptom score from the CBCL, parent’s highest education level, and family income. An additional analysis was also added that also used stimulant medication status (yes/no) as a covariate and so we examined stimulant medication status’ association with ADHD symptomatology. Additionally, framewise displacement from the relevant fMRI scan was used as a covariate and for structural MRI the average of framewise displacement from the fMRI scans was used. In structural MRI analyses, total intracranial volume was also used as covariate. To account for the large numbers of siblings and multiple data collection sites, family ID (used to denote sibling status) was modeled as a random effect nested inside a random effect of scanner in all mixed effects models^1^, as has been recommended^37^ and is standard on the ABCD Data Exploration and Analysis Portal (DEAP). This nested approach was used since all siblings in each family were collected at the same scanner site. In these analyses, the covariates were the independent variables and ADHD symptomatology was the dependent variable. Additionally, to investigate if working memory and inhibitory control were associated with ADHD symptomatology, we examined the association of behavioral performance on the EN-Back and Stop Signal tasks with attention symptoms. d′ (*z*(hit rate) − *z*(false alarm rate)) was used as the performance measure for the EN-Back tasks and stop signal reaction time was used as the performance metric for SST. In these analyses, the performance metrics were used as the independent variables, along with all fixed effect covariates, and ADHD symptomatology was used as the dependent variable.

#### Primary analyses: elastic net regression

Elastic net regression was used to build predictive models for each of the imaging modalities (structural MRI, EN-Back, SST, and MID) using the glmnet package in MATLAB R2018b. Separate models were built for each MRI paradigm (i.e., each of three fMRI task + sMRI), with all brain variables used as features (i.e., independent variables) and ADHD symptomatology used as the target (i.e., dependent variable). Three versions of the analysis were run, one in which covariates were not accounted for, one in which the ADHD symptomatology score was residualized so that its shared variance with the covariates was removed, and one in which covariates including medication status were residualized from ADHD symptomatology.

Initially, data were divided in an 80%/20% split, with the 80% used as a training/validation set for model building and the 20% used as a final external test set. Then, elastic net regression model training was conducted in a 5-fold internal cross-validation framework with 80% of the data used for model training and 20% of the data used as an internal validation set to assess the model’s performance out-of-sample. Regularization hyperparameter tuning was conducted through a further nesting of a 20-fold cross-validation within the 5-fold cross validation (see Supplemental Methods for details on the cross-validation framework). Prediction accuracy was measured in *R*^*2*^. For the internal cross-validation, each *R*^2^ represents the accuracy of predicting the internal validation set (the 5th fold) using the model built on the training set (folds 1–4); to confirm the model’s generalizability, another *R*^*2*^ was derived by predicting the external test set (i.e., 20% of total data) using the most successful of the 5 models built in the 5-fold cross-validation. See Supplemental Methods for more details on the elastic net cross-validation scheme.

#### Primary analysis: confirmatory univariate analyses

To aid with the interpretation of elastic net regression analyses, follow-up analyses were conducted testing the associations of ADHD symptomatology with each of the brain features from the best elastic net regression model from each modality. This was done with a separate mixed effects model for each brain feature, which was used as a fixed effect. Covariates were fixed effects, family and scanner were random effects, and ADHD symptomatology was the dependent variable. Since these analyses were confirming relationships of coefficients found in elastic net models, a threshold of *p* < 0.05 was used to indicate significance. Regions included in elastic net regression models were only considered as neural correlates of ADHD symptomatology if they were also associated in the same direction in confirmatory univariate analyses.

#### Secondary analysis: categorical analyses

Because of the non-normal distribution of ADHD symptomatology (see Supplemental Fig. 1), we also completed a supplementary analysis to ensure that results were not being driven by this distribution. For this analysis we created a categorical variable of ADHD symptomatology based on a tertile split of the variable (group 1: no symptoms, 0 on CBCL; group 2: low symptoms, 1–3 on CBCL; and group 3: high symptoms, ≥3 on the CBCL). The three ADHD symptomatology groups were roughly equal in size (see Supplemental Fig. 3). Then we repeated the primary analyses using this categorical ADHD symptomatology variable as the dependent variable. Additionally, to determine if continuous findings of ADHD symptomatology would generalize to a clinical diagnosis of ADHD, we also repeated the primary analysis using ADHD diagnosis from the K-SADS as the dependent variable (K-SADS measure described in Supplemental Methods). There were 727 participants with an ADHD diagnosis vs. 7745 controls with valid sMRI data (N.B., 124 participants from the primary analyses did not have K-SADS ADHD diagnosis data and were excluded from this analysis). See Supplemental Fig. 4 for visualization of distribution of K-SADS diagnosis.

## Results

### Preliminary mixed effect analyses

In the full sample, all demographic covariates but age were related to ADHD symptomatology (Table 2), with the largest effects being observed for internalizing symptoms (positive association with ADHD symptomatology), followed by medication status (being on medication to treat ADHD positively associated with ADHD symptomatology), and sex (being male positively associated with ADHD symptomatology). In-scanner motion on each of the three tasks was positively related to ADHD symptomatology and intracranial volume was negatively related to ADHD symptomatology. ADHD symptomatology was associated with worse performance on the EN-Back (*B* = −0.298, SE = 0.054, *t* = 5.53, *p* = 3.3E-8, *R*^*2*^ = 0.6%) and on the SST (*B* = 0.002, SE = 0.001, *t* = 2.69, *p* = 0.007, *R*^*2*^ = 0.1%).

**Table 2:** Associations of demographic covariates with attention deficit hyperactivity disorder symptoms.

Phenotype (IV)	*B*	SE	*p*	*R*^*2*^
Age	−0.004	0.005	0.450	0.000
Pubertal status	−0.105	0.049	0.031	0.001
Sex (male)	1.036	0.073	<0.001	0.023
Handedness	0.325	0.051	<0.001	0.005
Internalizing symptoms	0.323	0.006	<0.001	0.259
Parent’s highest education (high school)	−0.089	0.247	0.719	0.009
Parent’s highest education (some college)	0.116	0.221	0.598	0.009
Parent’s highest education (bachelor’s)	−0.301	0.222	0.175	0.009
Parent’s highest education (graduate)	−0.706	0.219	0.001	0.009
Child race (Black)	0.267	0.118	0.024	0.003
Child race (Asian)	−1.001	0.265	<0.001	0.003
Child race (other)	0.321	0.106	0.003	0.003
Parent’s income ($50,000–100,000)	−0.524	0.102	<0.001	0.009
Parent’s income (>$100,000)	−0.874	0.096	<0.001	0.009
Medication status	5.131	0.132	<0.001	0.150
EN-back motion	1.003	0.190	<0.001	0.005
SST motion	1.131	0.251	<0.001	0.004
MID motion	2.299	0.238	<0.001	0.015
Average motion	1.591	0.128	<0.001	0.018
Intracranial volume	0.027	0.036	0.458	0.000

Age, pubertal status, handedness, and internalizing symptoms were analyzed as continuous variables. Sex, parent’s education, parent’s income, child’s race, and medication status were analyzed as categorical variables. Sample size reflects sMRI analyses (which includes all subjects in fMRI analyses) for all rows except motion. For multi-category variables (e.g., race and income) *R*^*2*^ represents the variance explained by all dummy variables for a given category. For race “White” was used as the reference category; for income “<$50,000” was used as the reference category, for parent’s highest education “Less than a high school degree” was used as the reference category.

*B* unstandardized regression coefficient, *SE* standard error of regression coefficient, *t* t-statistic of regression coefficient, *p* alpha value of regression coefficient, *R*^2^ variance explained by that variable.

### Primary analysis

**Fig. 1:**
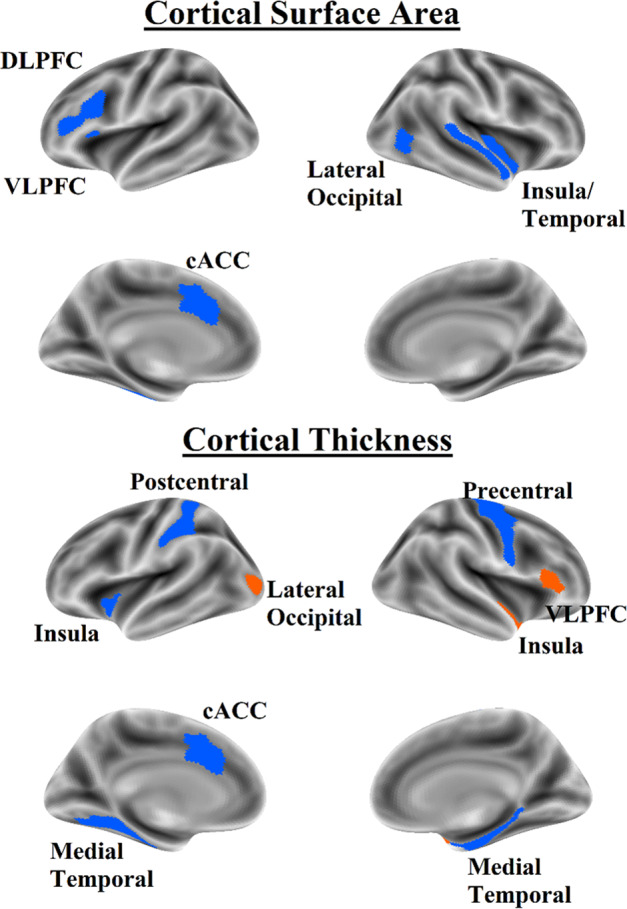
Brain map for sMRI features that predicted ADHD Symptomatology in the elastic net regression and were associated with ADHD symptomatology in univariate mixed effect models. Orange indicates positive coefficients; blue indicates negative coefficients. DLPFC dorsolateral prefrontal cortex, VLPFC ventrolateral prefrontal cortex, cACC caudal anterior cingulate cortex.

When not residualizing for the effects of potentially confounding variables, the best model for sMRI data explained 1.1% of the variance in ADHD symptomatology in the internal validation set, with some shrinkage when tested on the external test set (*R*^*2*^ = 0.8%; all model statistics in Table 3). However, sMRI elastic net models were less effective when ADHD symptomatology was residualized for confounding variables, with an *R*^*2*^ = 0.2% for the best model in the 5-fold cross-validation which explained 0% of the variance in the external test set. Likewise, when medication was included as a covariate the best sMRI model found an *R*^*2*^ = 0% for the best model in the 5-fold cross-validation which explained 0.1% of the variance in the external test set. In non-covaried analyses, different regional patterns emerged for CSA and CT. ADHD symptomatology was predicted by lower CSA in the DLPFC, VLPFC, ACC, insula, lateral temporal cortex, and lateral occipital cortex. In contrast, ADHD symptomatology was predicted by lower CT in the ACC, left insula, medial temporal cortex, precentral gyrus, and postcentral sulcus, and greater CT in the VLPFC, right insula, and lateral occipital cortex. The only region in which ADHD symptomatology was predicted by subcortical gray matter volume was the caudate, for which less gray matter volume predicted greater ADHD symptomatology. Regions identified in elastic net regression and confirmed in univariate analyses are shown in Fig. 1 and reported in Supplemental Table 3.

**Table 3:** Prediction accuracy (*R*^2^) for elastic net regression models.

Raw ADHD symptomatology	EN-back (%)	Structural MRI (%)	SST (%)	MID (%)
Model 1	−0.1	0.7	−1.2	−0.2
Model 2	2.0	0.8	−0.9	−0.3
Model 3	1.3	1.1	0.5	0.0
Model 4	−0.2	0.7	−0.1	0.1
Model 5	1.0	0.4	0.0	−0.1
Mean	0.8	0.7	0.3	−0.1
External test	1.9	0.8	0.1	0.1

Covs residualized ADHD symptomatology	EN-back (%)	Structural MRI (%)	SST (%)	MID (%)
Model 1	−0.5	0.1	−0.6	0.0
Model 2	1.2	0.2	−0.9	−0.1
Model 3	1.4	−0.3	0.1	−0.1
Model 4	−0.6	0.0	−0.1	0.0
Model 5	1.0	−0.4	0.3	0.0
Mean	0.5	−0.1	−0.2	0.0
External test	1.0	0.0	−0.3	0.1

Covs + medication residualized ADHD symptomatology	EN-back (%)	Structural MRI (%)	SST (%)	MID (%)
Model 1	−0.9	−0.3	−0.9	−0.2
Model 2	0.6	0.0	−0.6	−0.2
Model 3	1.0	−0.1	0.0	−0.1
Model 4	−0.9	−0.2	−0.1	0.0
Model 5	0.4	−0.2	−0.1	0.0
Mean	0.0	−0.2	−0.3	−0.1
External test	0.6	0.1	−0.1	0.0

Models 1–5 indicate the *R*^2^ of the models predicting the internal validation set in the training phase. “External Validation” indicates the *R*^2^ of the best model from the training phase being tested on the external test set. Full elaboration of the models can be found in Supplemental Tables.

*SST* stop signal task, MID monetary incentive delay task.

^a^Indicates model used no features (i.e., predicted intercept for all cases).

**Fig. 2:**
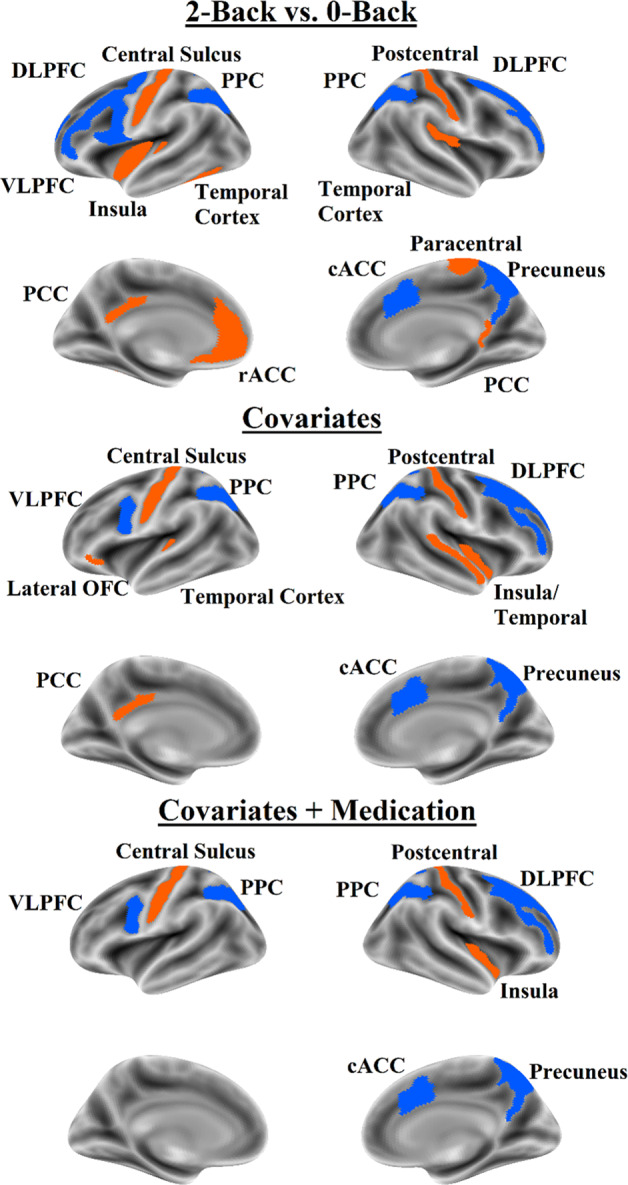
Brain map for fMRI features that predicted ADHD symptomatology in the elastic net regression and were associated with ADHD symptomatology in univariate mixed effect models. Orange indicates positive coefficients; blue indicates negative coefficients. Top panel = no covariate analysis, middle panel = analysis with covariates except medication status; bottom panel = all covariates including medication status. DLPFC dorsolateral prefrontal cortex, VLPFC ventrolateral prefrontal cortex, cACC caudal anterior cingulate cortex, rACC rostral anterior cingulate cortex, OFC orbitofrontal cortex, PPC posterior parietal cortex, PCC posterior cingulate cortex.

The best EN-Back model predicted ADHD symptomatology with accuracy of *R*^*2*^ = 2.0% in the internal validation set and *R*^*2*^ = 1.9% in the external test set. When predicting ADHD symptomatology with covariates residualized, the best EN-Back model predicted it with an accuracy of *R*^*2*^ = 1.4% in the internal validation set and with an accuracy of *R*^*2*^ = 1.0% in the external test set. When medication was added as a covariate, the best EN-Back model had an *R*^*2*^ = 0.4% on the internal validation and 0.6% on the external test set. In all three covariate schemes, ADHD symptomatology was predicted by lower activation in task positive regions including the DLPFC, VLPFC, caudal ACC, and PPC. ADHD symptomatology was also predicted by less deactivation in task negative regions including the central sulcus and postcentral gyrus and the inferior insula; when covariates were not considered the model also included less deactivation in the rostral ACC posterior cingulate cortex, lateral temporal cortex, and paracentral lobule. Regions identified in elastic net regression and confirmed in univariate analyses are shown in Fig. 2 and reported in Supplemental Table 4 (not accounting for covariates), Supplemental Table 5 (accounting for covariates except medication), and Supplemental Table 6 (accounting for covariates including medication).

The best model for SST predicted raw ADHD symptomatology with *R*^*2*^ = 0.5% in the internal validation set and with *R*^*2*^ = 0.1% in the external test set. The best model for SST predicting ADHD symptomatology with covariates residualized had an *R*^*2*^ = 0.1% on the internal validation set and an *R*^*2*^ = −0.03% on the external test set; in this analysis, negative *R*^*2*^ values means the best models were still worse than predicting the mean ADHD symptomatology score for all cases. When medication was added as a covariate, the best SST model had an *R*^*2*^ = 0% on the internal validation and −0.1% on the external test set. The best model for the MID predicted ADHD symptomatology with an *R*^*2*^ = 0.1% in the internal validation set, although this was consistent with the external test set (*R*^*2*^ = 0.1%). When covariates were residualized, the best MID model had an *R*^*2*^ = 0% on the internal validation set and *R*^*2*^ = 0.1% on the external test set. When medication was added as a covariate, the best MID model had an *R*^*2*^ = 0% on the internal validation set and *R*^*2*^ = 0% on the external test set. Given the poor predictive ability of the SST and MID models, we did not investigate their features.

### Secondary analysis: categorical analyses

Results of the elastic net regression and mixed effects modeling for categorical ADHD symptomatology using a tertile split of the CBCL were quite similar to the primary analyses of the study that used the CBCL ADHD symptomatology measure continuously. In short, with the categorical version of the CBCL the EN-Back predicted categorical ADHD symptomatology beyond covariates, with models using similar regions as those predicting continuous ADHD symptomatology. sMRI predicted categorical ADHD symptomatology but not beyond covariates. SST and MID did not predict categorical ADHD symptomatology. In contrast, elastic net analyses were not able to derive a model that robustly predicted ADHD diagnosis from the K-SADS ADHD diagnosis using any of the three task fMRI paradigms or sMRI. These results are reported in Supplemental Results, Supplemental Tables 7–9, and Supplemental Figs. 5 and 6.

## Discussion

The current study used elastic net regression with nested cross-validation to build models for predicting ADHD symptomatology out-of-sample using four sMRI and fMRI paradigms. Results indicate that the fMRI EN-Back working memory task was the most useful imaging paradigm to predict ADHD symptomatology even when accounting for numerous sociodemographic factors. Brain morphometry was able to predict ADHD symptomatology when sociodemographic factors were not accounted for, but not when covariates were accounted for. Even without accounting for sociodemographic factors, the best models for SST and MID were not effective at predicting ADHD symptomatology. Based on literature demonstrating individuals with ADHD symptomatology show poorer performance and different neural activation for working memory^38^, inhibitory control^9,11^, and reward processing^10^, we were surprised to see only working memory activation was able to predict ADHD symptomatology.

The features used by the best EN-Back models suggest that as ADHD symptomatology increases, activation in task positive regions (DLPFC, PPC, and caudal ACC) decreases and activation in task negative regions (VMPFC, posterior cingulate cortex, lateral temporal cortex, precentral, and postcentral gyri) increases. This is highly consistent with the existing literature, which has shown that during working memory tasks, individuals with ADHD show reduced activation in task positive regions^8,38,39^ and increased activation in task negative regions^40^.

The CSA features identified by the best sMRI model overlapped considerably with those in the EN-Back models; ADHD symptomatology was predicted by less CSA in the DLPFC, caudal ACC, and lateral temporal cortex. These findings converge with the largest prior study of the association of ADHD symptomatology and CSA in adolescents, which also found that ADHD symptomatology were linked to less CSA in the DLPFC and caudal ACC^14^. Regarding subcortical GMV, the caudate was the only region in which GMV appeared in the best elastic net regression model and was confirmed in univariate analysis to be associated with ADHD symptomatology. This is consistent with the largest examination of the correlates of ADHD for subcortical GMV in youths, which also found lower caudate volume in children with ADHD^15^. In the current study, ADHD symptomatology was predicted by lower CT in the caudal ACC, precentral and postcentral gyri, and medial temporal lobe, and higher CT in the VLPFC and medial and lateral occipital cortex, partially overlapping with the regions observed in the EN-Back model. Given that CT was not linked to ADHD symptomatology in the study by Bayard et al. and the literature on CT and ADHD symptomatology has not been consistent across small studies (see Lin and Roth^11^), we would describe the regional findings detailed here as preliminary.

While the relations of CSA and caudate GMV have been reproduced across studies when covariates are not accounted for (or are minimally accounted for), the question remains as to whether it is more appropriate to correct for demographic factors that likely are related to both ADHD symptomatology and brain structure and function and may play a causal role in their development. In the current study, accounting for age, sex, race, handedness, pubertal status, comorbid internalizing psychopathology, parental income, and parental education reduced the prediction accuracy of the elastic net regression considerably (best model *R*^*2*^ = 0.2% on internal validation set, 0.0% on external test set). Covariate use in the structural MRI literature is varied^41^ and it creates considerable ambiguities in interpreting findings; in the present findings, it is unclear if the factors proposed as covariates should be considered confounding third variables or meaningful and potentially causal factors. For example, it is very likely that growing up in impoverished circumstances affects the brain’s development and increases the risk of developing ADHD symptomatology^42,43^; consequently to remove variance in brain and ADHD symptomatology that is shared by socioeconomic status may serve to deflate the magnitude of the association between the two. While the field of cognitive neuroscience works towards a consensus on how best to handle covariates, we opted to report results both with and without covariates.

It is notable that neither the SST nor MID models were effective at predicting ADHD symptomatology. However, we do not think that the current results should be interpreted as definitive evidence that there are no differences in brain activation during inhibitory or reward tasks in individuals with ADHD. Future work might further interrogate these tasks for relationships with ADHD symptomatology. For example, there are alternate approaches to analyzing these tasks (e.g., functional connectivity, computational modeling) that may prove better for investigating how inhibitory control and reward processing differences in those with ADHD symptomatology manifest in the brain. Alternately, it is also possible that prior findings linking ADHD symptomatology to SST and MID were spurious or that differences in the neural correlates of inhibitory control and reward processing are limited to other age groups, as most prior studies examined older adolescents and adults.

### Considerations

A major consideration for the current study is the magnitude of the observed effect sizes. Even the best model predicting ADHD symptomatology explained just 2% of the variance out of sample – equivalent to a Pearson’s correlation of 0.14. According to Cohen’s heuristic, a correlation of 0.1 is considered small^44^. However, it has been noted that these guidelines understate the importance of associations between 0.1 and 0.2, as many relationships in day-to-day life that most would consider meaningful fall within this range^45^, such as the relationship of antihistamine use to allergy symptoms (Pearson’s *r* = 0.11), ibuprofen to headache severity (Pearson’s *r* = 0.14), combat exposure in the Vietnam war to likelihood of developing PTSD (Pearson’s *r* = 0.11), college grades to job performance (Pearson’s *r* = 0.14), and the critical ratings to a film’s box office success (Pearson’s *r* = 0.17)^46^. As larger datasets become available, it is becoming more apparent that prior small studies showing “large” associations between brain and behavior were much too optimistic^47^. It is also worth noting that small effect sizes found in the current study are consistent with other large MRI studies on the neurobiological correlates of ADHD. For example, a recent ENIGMA ADHD study found small effect sizes for differences in gray matter volume in individuals with ADHD (Cohen’s *d* ~ 0.1, equivalent to *R*^2^ = 2%)^15^. Likewise, a recent IMAGEN study found small effect sizes for associations of cortical thickness to ADHD symptoms in adolescents (*f*^2^ = 0.01, equivalent to *R*^2^ = 1%). The largest and most thorough machine learning study to examine ADHD in children (also using the ENIGMA dataset) found that their best model using sMRI predicted ADHD with an AUC = 0.67 (equivalent to *R*^2^ = 9%)^17^. This is slightly larger than the effects in the current study but is consistent when considering that these studies were comparing a clinical sample of individuals with an ADHD diagnosis and controls without ADHD symptomatology. There have not been any comparable and adequately powered studies with which to compare effect sizes to the current results for the EN-Back.

Another consideration is that of in-scanner motion. Children with ADHD symptomatology are known to be prone to fidgeting during MRI sessions and in the current study in-scanner motion explained 1–2% of the variance in ADHD symptomatology across fMRI scans. As a consequence, we made several attempts to account for in-scanner motion, such as using a stringent exclusion criteria for excess in-scanner motion (total framewise displacement >0.9 mm and volume censoring leaving fewer than 200 TRs of usable data) and covarying for in-scanner motion in all analyses. While this provides protection against the possibility that results are entirely a consequence of movement-induced scanner artifact, it also means that children with the most severe ADHD symptomatology were the most likely to be excluded, resulting in artificially truncated variance in ADHD symptomatology. This may contribute to the small effects seen in the current study and it is possible that effects may have been somewhat larger if these exclusions were not necessary to guard against artifact. As such, technical innovations in fMRI acquisition or processing that can reduce motion or its impacts on data would be tremendously helpful in the study of the neurobiology of ADHD symptomatology.

It is also worth considering the measure used to assess ADHD symptomatology. The CBCL is among the best validated parent-report measures of child ADHD symptomatology^48,49^; it shows excellent concordance with clinical interviews done by mental health professionals and convergent and discriminant validity with numerous other indicators of psychopathology. Increasingly, research suggests that ADHD is a dimensional construct and levels of attention problems that do not meet the diagnostic threshold for ADHD can have negative effects on individuals’ quality of life^21^. Given that the current study used a non-clinical sample, we think measuring ADHD continuously was the most appropriate approach. Notably, when we re-analyzed the data using ADHD diagnosis from the K-SADS as the target variable our elastic net approach was not able to build a model that could robustly predict ADHD diagnosis. We suspect that this was a result of the loss of statistical power from artificially binarizing a continuous phenomenon into imbalanced groups. However, we do recognize this as a limitation of the study, as our results cannot be directly generalized to a formal ADHD diagnosis, which (despite its flaws) does represent the dominate approach in modern clinical psychiatry. It is also a limitation that our current results do not speak to specific ADHD subtypes, but rather focus on ADHD symptomatology in the aggregate.

One further consideration is the medication status of the participants. Results were not affected substantially by including each participant’s medication status as a covariate, suggesting that medication was not the primary driver of differences in brain structure and function in individuals with ADHD symptomatology. This was expected based on prior literature, which suggested that medication effects would not represent a substantial confound to the current study. The largest previous study on this topic to date found no differences in morphometry between children with ADHD on and off stimulant medication^15^. Several meta-analyses of task fMRI differences in children with ADHD have found similar patterns of activation for children on and off medication^7,8^.

## Conclusion

Using elastic net regression with nested cross-validation, the current study found compelling evidence that ADHD symptomatology is associated with less activation during engagement of working memory in task positive regions of the brain and more activation in task negative regions, consistent with prior literature. The current study also confirmed prior work indicating that lower CSA and GMV of the caudate is associated with more ADHD symptomatology, though this was not the case after accounting for sociodemographic factors. Previously identified associations of activation during behavioral inhibition and reward processing were not confirmed in the current study.

## Supplementary information

Supplemental Materials

## Footnotes

1 Of note, when examined with a chi-square test, we found that the inclusion of family ID had no impact on the fit of the random effects model (comparison of models with and without family ID: *λ*^2^ = 0, *p* = 1). To ensure that its inclusion was not adversely affecting our analyses, we also ran all analyses using a simpler model that included only a random effect of scanner. Results using this approach were identical (within rounding), both for the elastic net and mixed effect model statistics.
