# Correction to: Multimethod investigation of the neurobiological basis of ADHD symptomatology in children aged 9-10: baseline data from the ABCD study

**DOI:** 10.1038/s41398-021-01320-y

**Published:** 2021-04-12

**Authors:** Max M. Owens, Nicholas Allgaier, Sage Hahn, DeKang Yuan, Matthew Albaugh, Shana Adise, Bader Chaarani, Joseph Ortigara, Anthony Juliano, Alexandra Potter, Hugh Garavan

**Affiliations:** Department of Psychiatry, University of Vermont, Burlington, VT 05401 USA

**Keywords:** Predictive markers, ADHD

Correction to: *Translational Psychiatry*

10.1038/s41398-020-01192-8 published online 18 January 2021

The original version of this article unfortunately contained a mistake. Due to a coding error, the final demographic table (Table 1) was reported incorrectly. The correct Table 1 can be found below. The authors apologize for the mistake. The original article has been corrected.

**Table 1:** Descriptive statistics on samples for each MRI paradigm.

	sMRI		EN-Back		SST		MID	
	Mean	SD	Mean	SD	Mean	SD	Mean	SD
Child age								
Months	119.18	7.46	119.61	7.53	119.53	7.54	119.42	7.52

	Total	%	Total	%	Total	%	Total	%
Child sex								
Female	4179	49%	2681	49%	2524	50%	2973	50%
Child race								
White	5917	69%	3878	72%	3596	72%	4174	70%
Black	1140	13%	592	11%	570	11%	712	12%
Asian	177	2%	111	2%	105	2%	118	2%
Other	1362	16%	836	15%	749	15%	955	16%
Child ethnicity								
Hispanic	1604	19%	997	18%	911	18%	1122	19%
Household income								
<$50K	2303	27%	1313	24%	1228	24%	1514	25%
$50K–$100K	2474	29%	1588	29%	1462	29%	1736	29%
>$100K	3819	44%	2516	46%	2330	46%	2709	45%
Parent education								
<HS diploma	283	3%	142	3%	136	3%	169	3%
HS diploma/GED	644	7%	352	6%	338	7%	421	7%
Some college	2153	25%	1303	24%	1197	24%	1465	25%
Bachelor	2336	27%	1547	29%	1434	29%	1675	28%
Post graduate degree	3180	37%	2073	38%	1915	38%	2229	37%

