# Supplementary material for: Multimethod investigation of the neurobiological basis of ADHD symptomatology in children aged 9-10: baseline data from the ABCD study
Source: Transl Psychiatry. 2021 Jan 18;11:64. doi: 10.1038/s41398-020-01192-8 (PMC7813832; doi:10.1038/s41398-020-01192-8)
Supplement: Supplementary file 1 — Supplemental Materials [file 41398_2020_1192_MOESM1_ESM.docx]

**Supplemental Methods**

*Kiddie Schedule for Affective Disorders and Schizophrenia (K-SADS)*

The K-SADS is a semi-structured interview to measure and diagnose current and past psychiatric disorders in children aged 6-18 years^1^. It was designed to promote early diagnosis by incorporating parent and child reports and relying on interviewer clinical judgement in determining if DSM criteria are met. The ADHD scale specifically has been shown to differentiate children with ADHD from children with bipolar disorder and has shown convergent validity with other measures of ADHD symptomatology^2^.

*Emotional N-Back Task*

The emotional N-Back task is a modified version of a traditional N-back task (e.g., as reported in that incorporates added elements of facial and emotional processing^3^. In this and other visual N-back tasks, participants are shown a series of stimuli and for each stimulus are asked to respond if that stimulus is the same or different from the one they saw *N* items ago (i.e., “*N* back”). The current emotional N-Back task had two conditions: a 2-back as its active condition and a 0-back as the baseline condition, which included similar visuo-motor demands but less working memory load. In the 0-back participants indicated if each stimulus matched a single target presented at the start of each block of trials. Responses on the 2-back and 0-back were input on a two-button keypad, with one button indicating the stimulus was a match and the other indicating no match.

The emotional N-Back consisted of two runs of eight blocks each. Blocks contained 10 trials lasting 2.5 seconds each and four fixation blocks lasting 15 seconds each. Blocks were preceded by 2.5 second instruction screen indicating the condition of the upcoming block. In a typical N-back task the stimuli are letters or numbers. In the emotional N-Back used in the current study, three of the four stimulus types were human faces, demonstrating happy, fearful, or neutral facial expressions, with facial expressions stimulus type being held constant within block. Faces were racially diverse and derived from two pre-existing collections: the NimStim emotional stimulus set^4^ and the Racially Diverse Affective Expressions (RADIATE) set of stimuli^5^. Additionally, images of places were used as a fourth stimulus type. Each stimulus type was used in four blocks (2 for 2-back and 2 for 0-back), resulting in 50 seconds of each condition for each stimulus type. d’ (*z*(hit rate) − *z*(false alarm rate)) was used as the performance measure for the N-back tasks. A schematic of what participants saw when completing tasks is shown in Figure 2. In the current study, fMRI contrasts used were regional activation during 2-Back working memory blocks relative to a baseline of activation during 0-Back control task.

*Monetary Incentive Delay Task (MID)*

A pre-existing MID was used, which included both anticipation and receipt of reward and loss^6,7^ In this task, participants attempt to win or avoid losing money by quickly responding to cue stimuli using a response box in their dominant hand. For “win” trials, participants could “win” or “not win” $5.00 or $.20 (US Dollars) depending on if they responded in the time allotted. For “lose” trials, they could either “not lose” or “lose” the same amounts by responding within the timeframe. In “neutral” trials participants completed the same action but with no money able to be won or lost. The specific sequence of each trial was as follows: participants were shown a cue denoting the trial type (2 seconds), with “win” trials shown in a pink circle, “lose” trials shown in a yellow square, and “neutral” trials shown in a blue triangle. Then participants viewed a fixation cross of jittered duration (1.5-4 seconds), followed by a signal to respond, denoted by a black shape that matched the trial cue. The duration of the response signal varied between trials and was determined dynamically for each participant based on their response speed on previous trials in order to achieve an approximately 60% win/40% loss balance. Participants then received written feedback (e.g., “You won $5”). Each run consisted of 50 consecutive trials, lasting a total of 5’42” per run. Two runs of the MID were completed. Participants were compensated based on their performance on the task with mean earnings being $21. In the current study, fMRI contrasts used were regional activation during reward anticipation relative to a baseline of neutral anticipation (anticipation of trials in which reward amount was set to $0) and regional activation during reward outcome relative to a baseline of neutral outcome (failure to win a reward).

*Stop Signal Task (SST)*

The SST included serial presentations of leftward and rightward facing arrows and instructed participants to indicate the direction of the arrows using a two-button response box (e.g., left button indicating leftward); this was the “go” signal. Participants were further instructed not to respond on trials where the left or right arrow was followed by an arrow pointing upward (the “stop signal”). Participants were instructed to respond as “quickly and accurately as possible.”

The SST had two runs, each including 180 trials of which 30 were “stop” trials. Each trial lasted 1 second. To ensure a balance of successful and failed stop trials, the interval between the “go” and “stop signal,” the stop-signal delay (SSD) varied dynamically based on a participant’s success on the prior stop trial, increasing by 50 ms had they successfully inhibited and decreasing by 50 ms if they had failed to inhibit. The performance variable used for the SST was stop signal reaction time (SSRT), which represents the duration required to inhibit a “go” response after a “stop signal” has been presented and functions as an index for inhibitory speed. For more information about the fMRI tasks, see Casey et al.^3^ In the current study, fMRI contrasts used were regional activation during successful stops and regional activation during failed stops, both were compared against a baseline of successful go trials.

*Magnetic Resonance Imaging Sequences*

Structural and functional magnetic resonance imaging (MRI) scans were conducted at sites across the United States using 26 different scanners from two vendors (Siemens and General Electric); there were also 3 sites using Philips scanners that were excluded from analyses due to an error in processing prior to their release. Scanner serial numbers were used to control for scanner effects in subsequent analyses. Scan protocols were harmonized across scanners and all used a 32-channel head coil. T1 and T2 scans were collected for structural MRI; these scans used a matrix of 256 x 256, FOV = 256 x 256, 176 slices, and voxel size of 1.0mm^3^. The T1 scan had TR=2500ms, TE=2.88ms, and lasted 7’12”. The T2 scan had TR=3200ms, TE=565ms, and lasted 6’35”. fMRI scans used multiband acceleration to a factor of 6 with TR=800ms, TE=30ms, matrix=90 x 90, FOV=216 x 216, 60 slices, voxel size of 2.4mm^3^, and flip angle=52°. Scan durations varied across the three tasks (see description of protocols below. See ^3^ Table 2, for detailed MRI/fMRI acquisition protocols for each platform. For all three tasks, total framewise displacement was used as an index of in-scanner motion; for the structural MRI scan estimated total intracranial volume was derived using standard Freesurfer procedures.

*Magnetic Resonance Imaging Data Processing*

MRI sequences are reported in Casey et al.^3^. sMRI data were preprocessed by the Data Analysis and Informatics Core (DAIC) of ABCD using FreeSurfer version 5.3^8^. These processing steps produced CT and CSA measures for each of the 74 Destrieux atlas^9^ regions of interest and GMV for nine subcortical regions plus the brainstem from the ASEG parcellation in FreeSurfer. The Destrieux atlas was used instead of the Desikan atlas, as it contains a larger, but still manageable number of regions (74 vs. the 34 in the Desikan atlas^10^, the other atlas used in the ABCD official data release) would be more effective in elastic net modeling. fMRI data were preprocessed by the DAIC using a multi-program pipeline, which is detailed in ^8^ and were summarized using the same 74 bilateral cortical regions and nine bilateral subcortical regions (plus the brainstem). The fMRI contrasts used for the SST were *incorrect stop – correct go* and *correct stop – correct go*; for the N-back the only contrast was *2-Back vs. 0-Back*; for the MID contrasts were *reward anticipation – neutral anticipation* and *reward outcome – no reward outcome*.

*Primary Analyses: Elastic Net Regression*

Elastic net regression was used to build predictive models for each of the imaging modalities (structural MRI, EN-back, SST, and MID) using the glmnet package in MATLAB R2018b. Separate models were built for each MRI task. For each task, all brain variables were used as features (i.e., independent variables) and attention problems was used as the target (i.e., dependent variable). Two versions of the analysis were run, one in which covariates were not accounted for (i.e., the target was raw attention problems) and one in which attention problems was residualized so that its shared variance with the covariates was removed (i.e., residualized attention problems was the target). Sociodemographic covariates were participants’ age, sex, race, pubertal status, handedness, internalizing symptom score from the CBCL, as well as their parent’s highest education level and family income. In the cross-validation approach used, 80% of the data was used for model training (i.e., the 5-fold internal cross-validation) and 20% of the data was used as an external test set to assess if the model’s performance in-sample was comparable to its performance on out-of-sample data. Prediction accuracy was measured in R^2^. For the internal cross-validation folds, each R^2^ represents the accuracy of predicting the validation set (the 5^th^ fold) using the model built on the training set (folds 1-4); for the external test set, one R^2^ was derived by predicting the external test set using the most successful of the 5 models built in the 5-fold cross-validation. Features were reported for all models with successful predictions on the external test set.

Regularization hyperparameter tuning was conducted through a further nesting of a 20-fold cross-validation within the 5-fold cross validation. This was done to determine the optimal combination of the elastic net regularization parameters alpha (α) and lambda (λ), with the goal of identifying the most generalizable combination of hyperparameters, as determined by performance on a set aside fold. These hyperparameters represent the ratio of ridge and lasso regularization (α) and the strength of the regularization overall (λ). Twenty values of α (.05 to 1.0 in increments of .05) and 100 values of λ (logarithmically spaced from .01 to an empirically determined maximum [see glmnet documentation for more details]) were tested. In the nested 20-fold cross validation, the training data (4/5 folds) of each of the five iterations of the outer 5-fold cross-validation was split into 20 folds. Within each of the 20 folds, 2000 combinations of α and λ were tested and the best combination selected. Then the combination which yields the best accuracy from all the folds was used in model building for that k-fold iteration.

**Supplemental References**

1 Kaufman J, Birmaher B, Brent D, Rao U, Flynn C, Moreci P *et al.* Schedule for affective disorders and schizophrenia for school-age children-present and lifetime version (K-SADS-PL): Initial reliability and validity data. *J Am Acad Child Adolesc Psychiatry* 1997; **36**: 980–988.

2 Geller B, Warner K, Williams M, Zimerman B. Prepubertal and young adolescent bipolarity versus ADHD: Assessment and validity using the WASH-U-KSADS, CBCL and TRF. *J Affect Disord* 1998. doi:10.1016/S0165-0327(98)00176-1.

3 Casey BJ, Cannonier T, Conley MI, Cohen AO, Barch DM, Heitzeg MM *et al.* The Adolescent Brain Cognitive Development (ABCD) study: Imaging acquisition across 21 sites. *Dev Cogn Neurosci* 2018; **32**: 43–54.

4 Tottenham N, Tanaka JW, Leon AC, McCarry T, Nurse M, Hare TA *et al.* The NimStim set of facial expressions: Judgments from untrained research participants. *Psychiatry Res* 2009. doi:10.1016/j.psychres.2008.05.006.

5 Conley MI, Dellarco D V., Rubien-Thomas E, Cohen AO, Cervera A, Tottenham N *et al.* The racially diverse affective expression (RADIATE) face stimulus set. *Psychiatry Res* 2018. doi:10.1016/j.psychres.2018.04.066.

6 Knutson B, Westdorp A, Kaiser E, Hommer D. FMRI visualization of brain activity during a monetary incentive delay task. *Neuroimage* 2000. doi:10.1006/nimg.2000.0593.

7 Yau WYW, Zubieta JK, Weiland BJ, Samudra PG, Zucker RA, Heitzeg MM. Nucleus accumbens response to incentive stimuli anticipation in children of alcoholics: Relationships with precursive behavioral risk and lifetime alcohol use. *J Neurosci* 2012. doi:10.1523/JNEUROSCI.1390-11.2012.

8 Hagler DJ, Hatton S, Cornejo MD, Makowski C, Fair DA, Dick AS *et al.* Image processing and analysis methods for the Adolescent Brain Cognitive Development Study. *Neuroimage* 2019; **202**: 116091.

9 Destrieux C, Fischl B, Dale A, Halgren E. Automatic parcellation of human cortical gyri and sulci using standard anatomical nomenclature. *Neuroimage* 2010; **53**: 1–15.

10 Desikan RS, Segonne F, Fischl B, Quinn BT, Dickerson BC, Blacker D *et al.* An automated labeling system for subdividing the human cerebral cortex on MRI scans into gyral based regions of interest. *Neuroimage* 2006; **31**: 968–980.


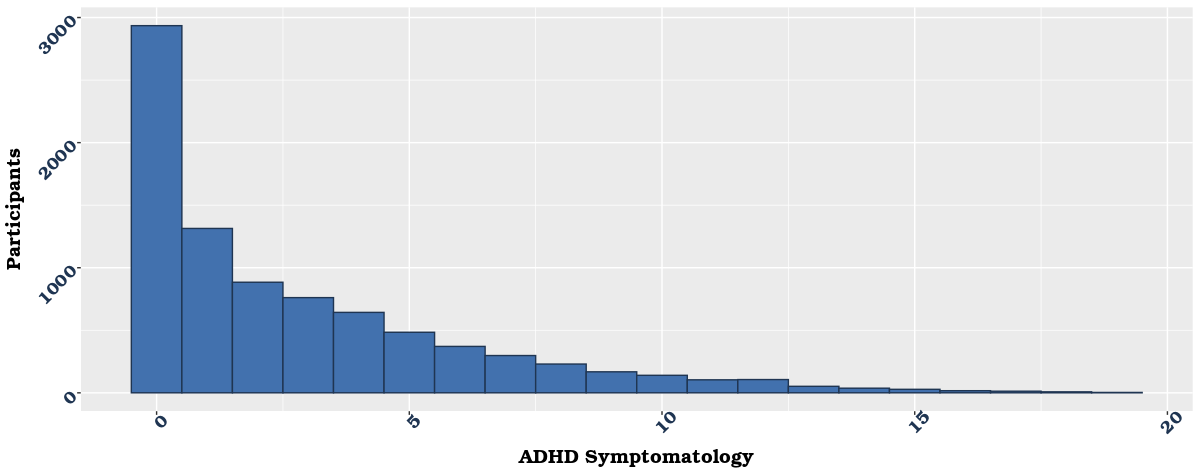
**Supplemental Figure 1.** Distribution of attention problems. N.B. - figure uses sample from structural MRI analyses.

**Supplemental Figure 2.** Schematic of Tasks.


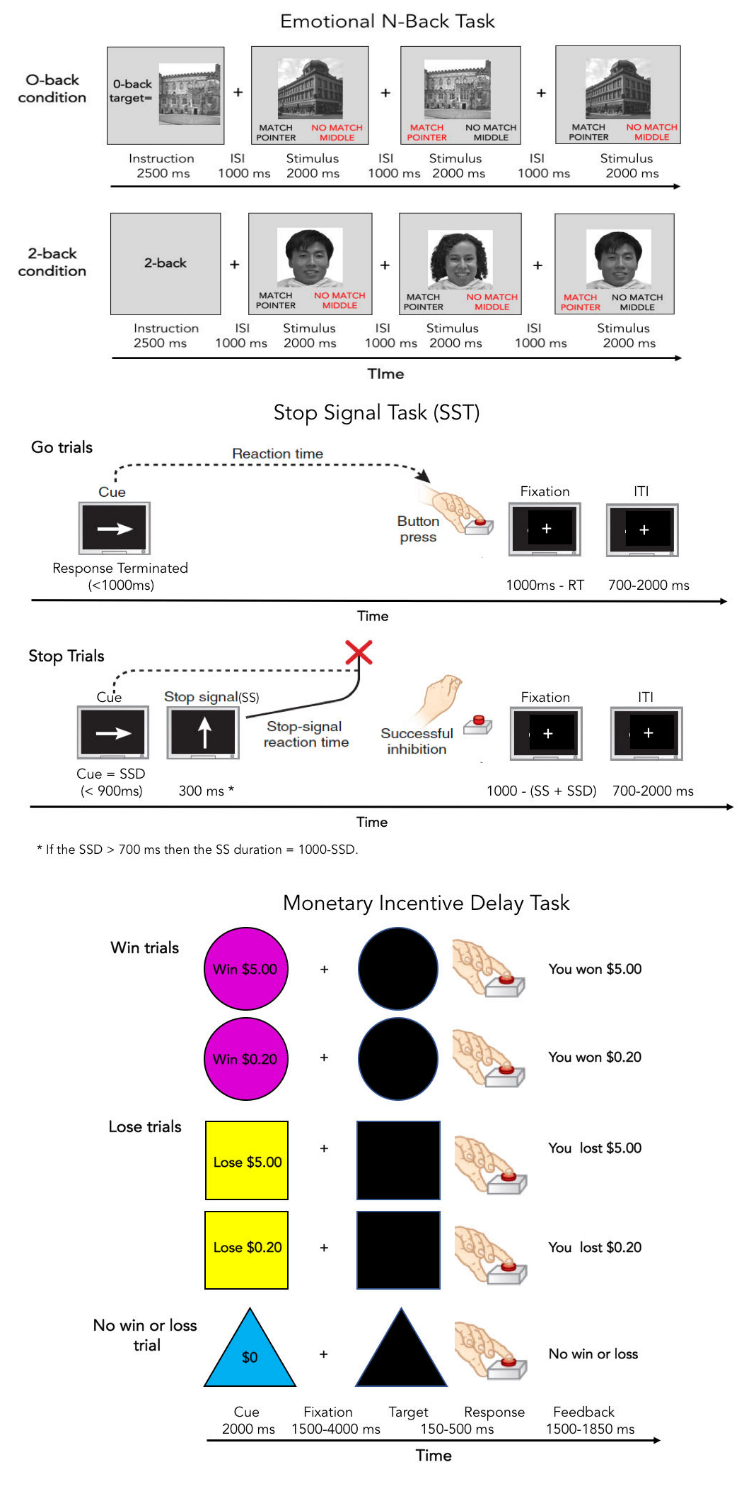


**Supplemental Figure 3.** Frequency of ADHD groupings from CBCL. N.B. - figure uses sample from structural MRI analyses.


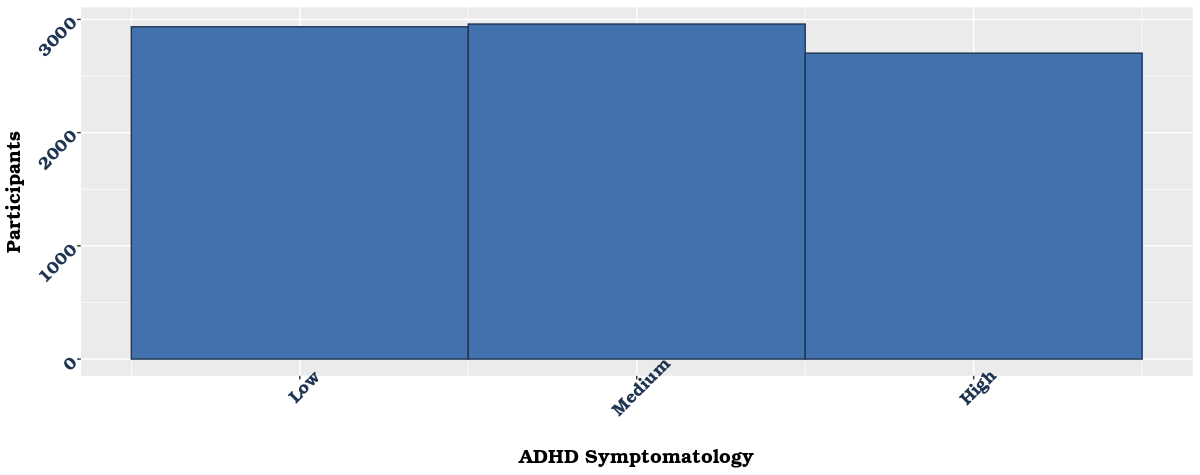


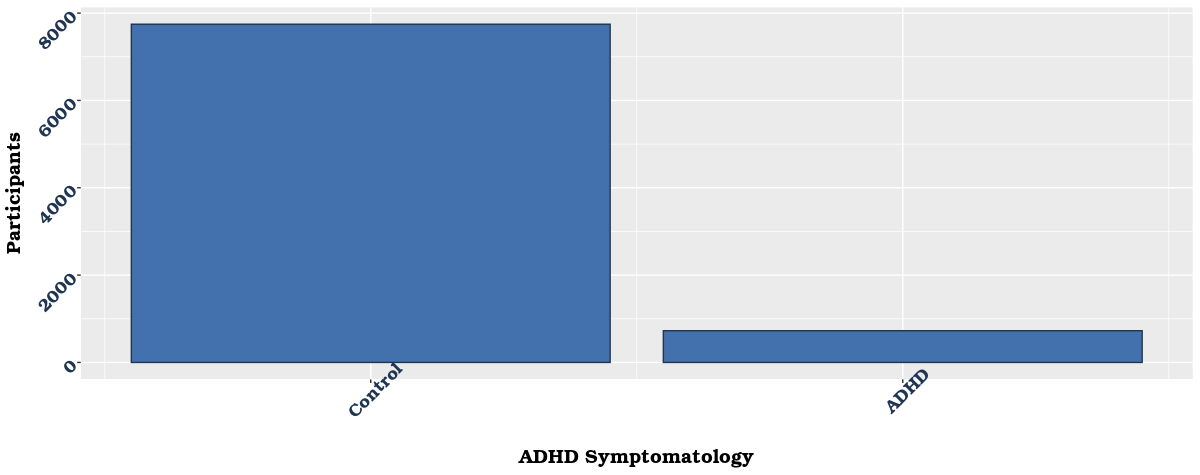
**Supplemental Figure 4.** Frequency of ADHD diagnosis. N.B. - figure uses sample from structural MRI analyses.

**Supplemental Figure 5.** Regions for which SMRI predicted/was associated with categorical ADHD Symptomatology when not considering covariates.


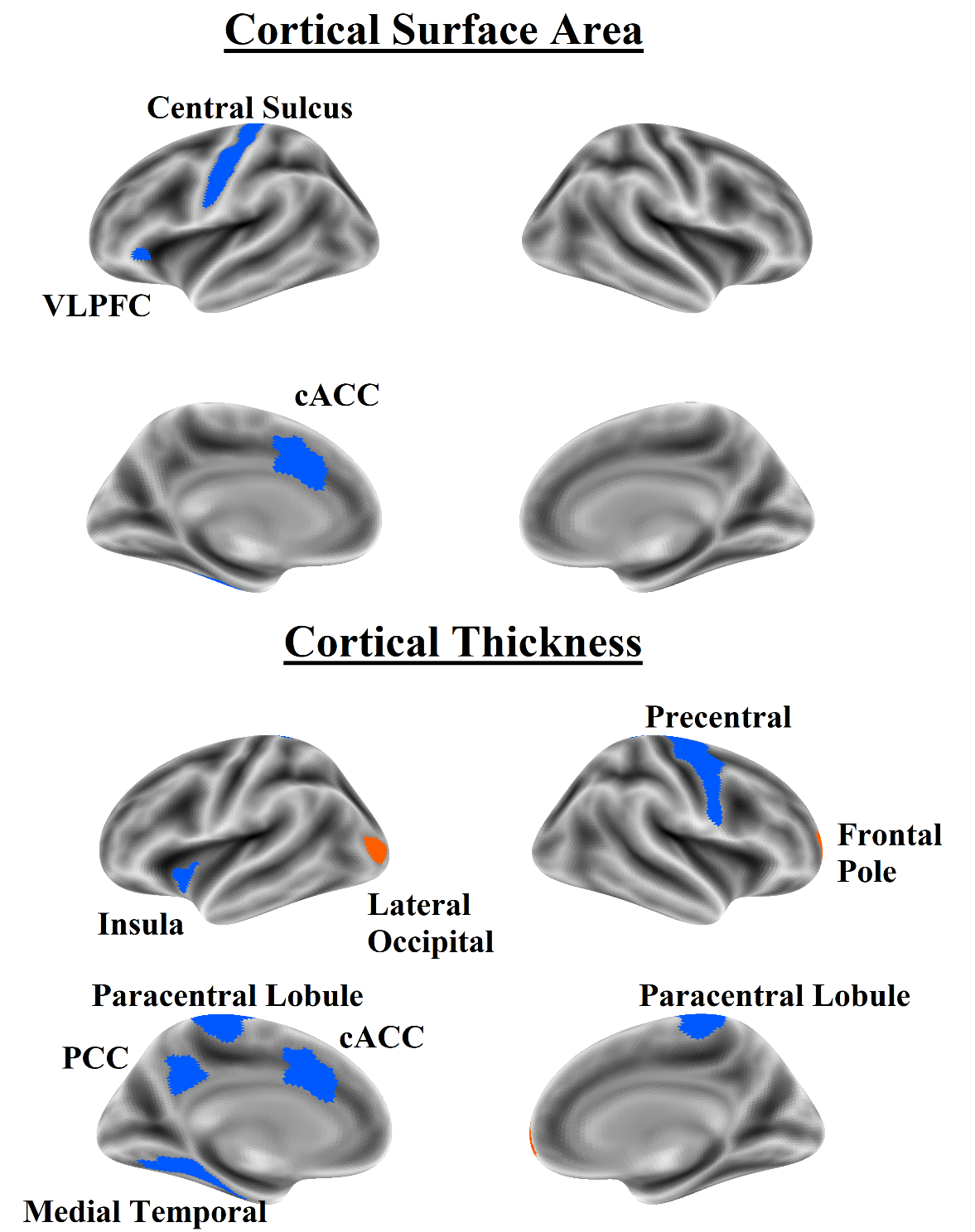


**Supplemental Figure 6.** Regions for which 2-Back activation (relative to 0-Back baseline) predicted/was associated with categorical ADHD Symptomatology when not considering covariates.


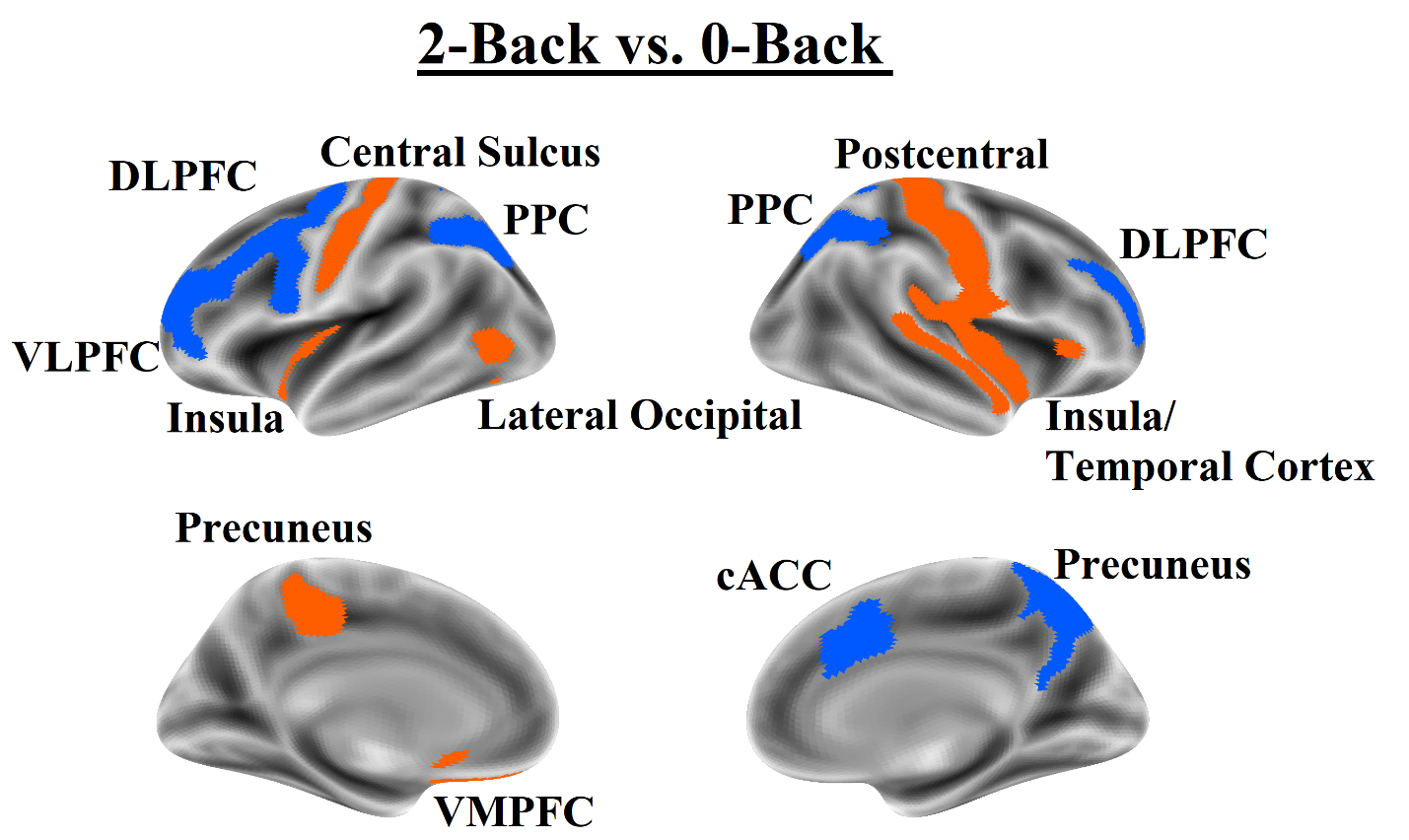


**Supplemental Table 1.** Table of subject exclusions across quality control steps.

|  | SMRI | MID | NBACK | SST |
| --- | --- | --- | --- | --- |
| Complete ROI Data | 11526 | 9073 | 8808 | 8873 |
| SMRI QC | 11066 | 8804 | 8558 | 8622 |
| FMRI QC | 11066 | 7481 | 6722 | 7002 |
| Phillips Scanner Removal | 11065 | 6561 | 5921 | 6122 |
| Missing CBCL | 11055 | 6558 | 5920 | 6121 |
| Missing Covariates | 8596 | 5959 | 5417 | 5580 |
| SST Glitch | 8596 | 5959 | 5417 | 5020 |

**Supplemental Table 2.**  Medications included in stimulant variable medication status variable.

| **Medication Name** |
| --- |
| Adderall |
| Concerta |
| Methylphenidate |
| Ritalin |
| Focalin |
| Strattera |
| Amphetamine |
| Quillivant |
| Guanfacine |
| Evekeo |
| Atomoxetine |
| Lisdexamfetamine |
| Dexedrine |
| Dynavel |
| Adzenys |
| Metadate |
| Kapvay |
| Clonidine |
| Intuniv |
| Daytrana |
| Methylin |
| Dextrostat |
| Zenzedi |
| Tenex |
| Catapres |
| Aptensio |
| Cotempla |
| Daytrana |
| Quillichew |
| Bupropion |
| Wellbutrin |
| Norpramin |
| Desipramine |
| Impiprmine |
| Tofranil |
| Nortriptyline |
| Aventyl |
| Pamelor |

**Supplemental Table 3.** Regions for which sMRI predicted/was associated with ADHD Symptomatology when not considering covariates. B_LME_= coefficient from mixed effects model; SE= standard error of coefficient from mixed effect model; P = alpha value for mixed effect coefficient; R^2^=variance explained by mixed effect coefficient; B_EN_ = coefficient in elastic net regression model.

| ROI | B_LME_ | SE | p | R^2^ | B_EN_ |
| --- | --- | --- | --- | --- | --- |
| Cortical Thickness Right Superior Precentral Sulcus | -1.084 | 0.273 | 7.4E-05 | 0.002 | -0.017 |
| Cortical Thickness Left Medial Occipito-Temporal Sulcus | -1.141 | 0.290 | 8.2E-05 | 0.002 | -0.026 |
| Cortical Thickness Right Precentral Gyrus | -0.801 | 0.208 | 1.2E-04 | 0.002 | -0.020 |
| Cortical Surface Area Left Middle-Anterior Cingulate | -0.001 | 0.000 | 4.3E-04 | 0.001 | -0.008 |
| Cortical Surface Area Left Anterior Transverse Collateral Sulcus | -0.001 | 0.000 | 0.001 | 0.001 | -0.002 |
| Cortical Surface Area Right Lateral Superior Temporal Gyrus | -0.001 | 0.000 | 0.001 | 0.001 | -0.013 |
| Cortical Surface Area Left Anterior Lateral Sulcus | -0.002 | 0.001 | 0.001 | 0.001 | -0.004 |
| Gray Matter Volume Right Caudate | 0.000 | 0.000 | 0.002 | 0.001 | -0.008 |
| Cortical Thickness Left Middle-Anterior Cingulate | -0.801 | 0.274 | 0.003 | 0.001 | -0.026 |
| Cortical Surface Area Right Inferior Circular Sulcus Insula | -0.001 | 0.000 | 0.004 | 0.001 | -0.008 |
| Cortical Surface Area Left Inferior Frontal Sulcus | 0.000 | 0.000 | 0.004 | 0.001 | -0.015 |
| Cortical Thickness Right Parahippocampal Gyrus | -0.460 | 0.163 | 0.005 | 0.001 | -0.012 |
| Cortical Thickness Left Postcentral Sulcus | -0.807 | 0.295 | 0.006 | 0.001 | -0.013 |
| Cortical Thickness Left Middle Occipital Sulcus & Lunatus Sulcus | 0.673 | 0.250 | 0.007 | 0.001 | 0.035 |
| Cortical Surface Area Right Anterior Occipital Sulcus | -0.001 | 0.000 | 0.014 | 0.001 | -0.002 |
| Cortical Thickness Right Planum Polare Superior Temporal Gyrus | 0.392 | 0.164 | 0.017 | 0.001 | 0.033 |
| Cortical Thickness Right Triangular Inferior Frontal Gyrus | 0.470 | 0.214 | 0.028 | 0.001 | 0.015 |
| Cortical Thickness Left Short Insular Gyri | -0.384 | 0.181 | 0.034 | 0.001 | -0.007 |

**Supplemental Table 4.** Regions for which 2-Back activation (relative to 0-Back baseline) predicted/was associated with ADHD Symptomatology when not considering covariates. B_LME_= _c_oefficient from mixed effects model; SE= standard error of coefficient from mixed effect model; T= t-statistic from mixed effect coefficient; P = alpha value for mixed effect coefficient; R^2^=variance explained by mixed effect coefficient; B_EN_ = coefficient in elastic net regression model.

| ROI | B_LME_ | SE | p | R^2^ | B_EN_ |
| --- | --- | --- | --- | --- | --- |
| 2-Back Activation Left Intraparietal Sulcus | -1.017 | 0.208 | 1.0E-06 | 0.004 | -0.022 |
| 2-Back Activation Right Intraparietal Sulcus | -0.945 | 0.211 | 7.9E-06 | 0.004 | -0.012 |
| 2-Back Activation Left Inferior Precentral Sulcus | -0.873 | 0.197 | 9.7E-06 | 0.004 | -0.004 |
| 2-Back Activation Right Middle-Anterior Cingulate | -1.041 | 0.253 | 3.8E-05 | 0.003 | -0.026 |
| 2-Back Activation Left Central Sulcus | 0.898 | 0.223 | 5.6E-05 | 0.003 | 0.015 |
| 2-Back Activation Right Middle Frontal Sulcus | -0.725 | 0.181 | 6.2E-05 | 0.003 | -0.006 |
| 2-Back Activation Right Superior Frontal Sulcus | -0.938 | 0.234 | 6.4E-05 | 0.003 | -0.015 |
| 2-Back Activation Right Precuneus | -0.851 | 0.214 | 6.8E-05 | 0.003 | -0.014 |
| 2-Back Activation Left Middle Frontal Gyrus | -0.828 | 0.210 | 8.2E-05 | 0.003 | -0.007 |
| 2-Back Activation Left Suborbital Sulcus | 0.342 | 0.091 | 1.8E-04 | 0.003 | 0.004 |
| 2-Back Activation Left Superior Precentral Sulcus | -0.884 | 0.245 | 3.0E-04 | 0.002 | -0.028 |
| 2-Back Activation Left Long Insular Gyrus & Central Sulcus Insula | 0.817 | 0.236 | 0.001 | 0.002 | 0.004 |
| 2-Back Activation Right Posterior Ramus | 0.721 | 0.211 | 0.001 | 0.002 | 0.002 |
| 2-Back Activation Right Cortex Cerebellum | -0.527 | 0.160 | 0.001 | 0.002 | -0.007 |
| 2-Back Activation Left Lateral Orbital Sulcus | -0.416 | 0.129 | 0.001 | 0.002 | -0.013 |
| 2-Back Activation Left Transverse Temporal Sulcus | 0.539 | 0.174 | 0.002 | 0.002 | 0.017 |
| 2-Back Activation Right Postcentral Gyrus | 0.694 | 0.237 | 0.003 | 0.002 | 0.014 |
| 2-Back Activation Left Subcallosal Area | 0.379 | 0.131 | 0.004 | 0.002 | 0.004 |
| 2-Back Activation Left Inferior Segment Circular Sulcus Insula | 0.683 | 0.256 | 0.008 | 0.001 | <0.001 |
| 2-Back Activation Left Anterior Cingulate | 0.537 | 0.219 | 0.014 | 0.001 | 0.008 |
| 2-Back Activation Right Paracentral Lobule & Sulcus | 0.516 | 0.217 | 0.017 | 0.001 | 0.006 |
| 2-Back Activation Right Posterior-Ventral Cingulate | 0.318 | 0.136 | 0.020 | 0.001 | 0.021 |
| 2-Back Activation Left Opercular Inferior Frontal Gyrus | -0.521 | 0.240 | 0.030 | 0.001 | -0.004 |
| 2-Back Activation Left Posterior-Dorsal Cingulate | 0.513 | 0.240 | 0.033 | 0.001 | 0.010 |
| 2-Back Activation Left Lateral Occipito-Temporal Sulcus | 0.465 | 0.218 | 0.033 | 0.001 | 0.027 |

**Supplemental Table 5.** Regions for which 2-Back activation predicted/was associated with ADHD Symptomatology when considering covariates. B_LME_= _c_oefficient from mixed effects model; SE= standard error of coefficient from mixed effect model; T= t-statistic from mixed effect coefficient; P = alpha value for mixed effect coefficient; R2=variance explained by mixed effect coefficient; B_EN_ = coefficient in elastic net regression model.

| ROI | B_LME_ | SE | p | R^2^ | B_EN_ |
| --- | --- | --- | --- | --- | --- |
| 2-Back Activation Left Intraparietal Sulcus | -0.841 | 0.177 | 2.0E-06 | 0.004 | -0.034 |
| 2-Back Activation Right Intraparietal Sulcus | -0.812 | 0.180 | 6.9E-06 | 0.004 | -0.013 |
| 2-Back Activation Left Inferior Precentral Sulcus | -0.707 | 0.167 | 2.5E-05 | 0.003 | -0.016 |
| 2-Back Activation Right Precuneus | -0.712 | 0.183 | 9.9E-05 | 0.003 | -0.008 |
| 2-Back Activation Left Central Sulcus | 0.717 | 0.189 | 1.5E-04 | 0.003 | 0.031 |
| 2-Back Activation Right Superior Frontal Sulcus | -0.753 | 0.200 | 1.7E-04 | 0.003 | -0.008 |
| 2-Back Activation Right Middle Frontal Gyrus | -0.569 | 0.167 | 0.001 | 0.002 | -0.006 |
| 2-Back Activation Right Middle-Anterior Cingulate | -0.716 | 0.215 | 0.001 | 0.002 | -0.020 |
| 2-Back Activation Left Transverse Temporal Sulcus | 0.461 | 0.147 | 0.002 | 0.002 | 0.010 |
| 2-Back Activation Right Cortex Cerebellum | -0.396 | 0.135 | 0.003 | 0.002 | -0.005 |
| 2-Back Activation Left Brain Stem | -0.553 | 0.202 | 0.006 | 0.001 | -0.008 |
| 2-Back Activation Right Postcentral Gyrus | 0.547 | 0.200 | 0.006 | 0.001 | 0.005 |
| 2-Back Activation Right Inferior Insula | 0.560 | 0.210 | 0.008 | 0.001 | 0.008 |
| 2-Back Activation Right Lateral Aspect Superior Temporal Gyrus | 0.486 | 0.226 | 0.031 | 0.001 | 0.016 |
| 2-Back Activation Left Orbital Inferior Frontal Gyrus | 0.248 | 0.123 | 0.044 | 0.001 | <0.001 |
| 2-Back Activation Left Posterior-Dorsal Cingulate | 0.409 | 0.203 | 0.044 | 0.001 | 0.021 |

**Supplemental Table 6.** Regions for which 2-Back activation predicted/was associated with ADHD Symptomatology when considering covariates plus medication as an additional covariate. B_LME_= _c_oefficient from mixed effects model; SE= standard error of coefficient from mixed effect model; T= t-statistic from mixed effect coefficient; P = alpha value for mixed effect coefficient; R^2^=variance explained by mixed effect coefficient; B_EN_ = coefficient in elastic net regression model.

| ROI | B_LME_ | SE | p | R^2^ | B_EN_ |
| --- | --- | --- | --- | --- | --- |
| 2-Back Activation Left Intraparietal Sulcus | -0.720 | 0.163 | 0.000 | 0.004 | -0.020 |
| 2-Back Activation Right Intraparietal Sulcus | -0.746 | 0.167 | 0.000 | 0.004 | -0.015 |
| 2-Back Activation Right Precuneus | -0.634 | 0.169 | 0.000 | 0.003 | -0.015 |
| 2-Back Activation Left Inferior Precentral Sulcus | -0.582 | 0.155 | 0.000 | 0.003 | -0.009 |
| 2-Back Activation Right Superior Frontal Sulcus | -0.621 | 0.185 | 0.001 | 0.002 | -0.010 |
| 2-Back Activation Right Middle Frontal Gyrus | -0.488 | 0.154 | 0.002 | 0.002 | -0.002 |
| 2-Back Activation Left Central Sulcus | 0.525 | 0.174 | 0.003 | 0.002 | 0.018 |
| 2-Back Activation Right Middle-Anterior Cingulate | -0.536 | 0.199 | 0.007 | 0.001 | -0.008 |
| 2-Back Activation Right Inferior Insula | 0.417 | 0.194 | 0.032 | 0.001 | 0.010 |
| 2-Back Activation Right Postcentral Gyrus | 0.391 | 0.185 | 0.035 | 0.001 | <0.001 |

**Supplementary Table 7.** R^2^ values from elastic net regression of categorical ADHD symptomatology (high/medium/low) and of ADHD diagnosis from the KSADS. For ADHD diagnosis a logistic elastic net regression was used yielding AUC, which was then converted to R^2^ using https://www.escal.site/; in the Table, AUC is reported in parentheses for ADHD diagnosis.

| Categorical ADHD Symptomatology | EN-Back | Structural MRI | SST | MID |
| --- | --- | --- | --- | --- |
| Model 1 | 0.6% | 0.6% | -0.3% | 0.2% |
| Model 2 | 1.3% | 0.9% | -0.9% | -0.3% |
| Model 3 | 1.5% | 0.7% | 0.2% | 0.0% |
| Model 4 | 0.2% | 0.5% | -0.2% | 0.2% |
| Model 5 | 1.2% | 0.1% | 0.2% | -0.4% |
| Mean | 1.0% | 0.6% | -0.2% | -0.1% |
| External Test | 1.9% | 0.7% | 0.2% | 0.0% |
| ADHD Diagnosis | **EN-Back** | **Structural MRI** | **SST** | **MID** |
| Model 1 | 0.4% (53.4%) | 0.1% (51.5%) | 0.0% (50.2%) | -0.8% (44.8%) |
| Model 2 | 4.0% (61.4%) | 0.8% (55.0%) | 0.0% (50.0%) | -0.3% (46.8%) |
| Model 3 | 2.2% (58.4%) | 0.8% (55.0%) | 0.0% (50.0%) | 0.0% (49.9%) |
| Model 4 | 0.3% (53.2%) | 0.5% (54.0%) | 0.0% (49.0%) | 0.0% (50.0%) |
| Model 5 | 1.3% (56.4%) | 0.0% (50.0%) | 0.0% (50.0%) | 0.0% (50.0%) |
| Mean | 1.1% (56.6%) | 0.3% (53.2%) | 0.0% (49.9%) | -0.1% (48.3%) |
| External Test | 0.2% (52.6%) | 0.1% (52.0%) | 0.0% (49.3%) | 0.0% (50.0%) |

**Supplemental Table 8.** Regions for which sMRI predicted/was associated with categorical ADHD Symptomatology when not considering covariates. B_LME_= coefficient from mixed effects model; SE= standard error of coefficient from mixed effect model; P = alpha value for mixed effect coefficient; R^2^=variance explained by mixed effect coefficient; B_EN_ = coefficient in elastic net regression model.

| ROI | B_LME_ | SE | p | R^2^ | B_EN_ |
| --- | --- | --- | --- | --- | --- |
| Cortical Thickness Left Medial Occipito-Temporal Sulcus | -0.292 | 0.069 | 2.40E-05 | 0.002 | -0.021 |
| Cortical Thickness Left Paracentral Lobule & Sulcus | -0.195 | 0.055 | 3.40E-04 | 0.001 | -0.011 |
| Cortical Thickness Right Precentral Gyrus | -0.178 | 0.050 | 3.40E-04 | 0.001 | -0.018 |
| Cortical Thickness Right Paracentral Lobule & Sulcus | -0.191 | 0.054 | 4.20E-04 | 0.001 | -0.002 |
| Cortical Surface Area Left Middle-Anterior Cingulate | 0.000 | 0.000 | 0.001 | 0.001 | -0.016 |
| Cortical Thickness Left Middle-Anterior Cingulate | -0.219 | 0.065 | 0.001 | 0.001 | -0.033 |
| Cortical Thickness Right Superior Precentral Sulcus | -0.212 | 0.065 | 0.001 | 0.001 | -0.006 |
| Cortical Surface Area Left Anterior Transverse Collateral Sulcus | 0.000 | 0.000 | 0.002 | 0.001 | -0.019 |
| Cortical Surface Area Left Central Sulcus | 0.000 | 0.000 | 0.013 | 0.001 | -0.001 |
| Cortical Thickness Transverse Frontopolar Gyri & Sulci | 0.103 | 0.043 | 0.018 | 0.001 | 0.013 |
| Cortical Thickness Left Short Insular Gyri | -0.097 | 0.043 | 0.026 | 0.001 | -0.008 |
| Cortical Thickness Left Middle Occipital Sulcus & Lunatus Sulcus | 0.129 | 0.060 | 0.031 | 0.001 | 0.018 |
| Cortical Thickness Left Subparietal Sulcus | -0.132 | 0.064 | 0.039 | <0.001 | -0.006 |
| Cortical Surface Area Left Horizontal Ramus of Anterior Lateral Sulcus | 0.000 | 0.000 | 0.042 | <0.001 | -0.003 |

**Supplemental Table 9.** Regions for which 2-Back activation (relative to 0-Back baseline) predicted/was associated with categorical ADHD Symptomatology when not considering covariates. B_LME_= _c_oefficient from mixed effects model; SE= standard error of coefficient from mixed effect model; T= t-statistic from mixed effect coefficient; P = alpha value for mixed effect coefficient; R2=variance explained by mixed effect coefficient; B_EN_ = coefficient in elastic net regression model.

| ROI | B_LME_ | SE | p | R^2^ | B_EN_ |
| --- | --- | --- | --- | --- | --- |
| 2-Back Activation Left Long Insular Gyrus & Central Sulcus Insula | 0.281 | 0.058 | 1.40E-06 | 0.004 | 0.027 |
| 2-Back Activation Left Intraparietal Sulcus | -0.224 | 0.051 | 1.20E-05 | 0.004 | -0.024 |
| 2-Back Activation Left Central Sulcus | 0.239 | 0.055 | 1.40E-05 | 0.003 | 0.021 |
| 2-Back Activation Right Posterior Ramus | 0.218 | 0.052 | 2.90E-05 | 0.003 | 0.017 |
| 2-Back Activation Right Intraparietal Sulcus | -0.207 | 0.052 | 7.30E-05 | 0.003 | -0.012 |
| 2-Back Activation Right Subcentral Gyrus & Sulcus | 0.212 | 0.054 | 8.90E-05 | 0.003 | 0.013 |
| 2-Back Activation Left Inferior Precentral Sulcus | -0.190 | 0.049 | 9.40E-05 | 0.003 | -0.021 |
| 2-Back Activation Right Central Sulcus | 0.216 | 0.056 | 1.10E-04 | 0.003 | 0.012 |
| 2-Back Activation Right Precuneus | -0.179 | 0.053 | 0.001 | 0.002 | -0.006 |
| 2-Back Activation Left Middle Frontal Gyrus | -0.171 | 0.052 | 0.001 | 0.002 | -0.004 |
| 2-Back Activation Right Inferior Segment Circular Sulcus Insula | 0.196 | 0.061 | 0.001 | 0.002 | 0.004 |
| 2-Back Activation Right Long Insular Gyrus & Central Sulcus Insula | 0.179 | 0.056 | 0.002 | 0.002 | <0.001 |
| 2-Back Activation Right Postcentral Gyrus | 0.181 | 0.058 | 0.002 | 0.002 | 0.004 |
| 2-Back Activation Right Middle Frontal Sulcus | -0.135 | 0.045 | 0.002 | 0.002 | -0.012 |
| 2-Back Activation Left Subcallosal Area | 0.095 | 0.032 | 0.003 | 0.002 | 0.01 |
| 2-Back Activation Right Cortex Cerebellum | -0.115 | 0.039 | 0.004 | 0.002 | -0.009 |
| 2-Back Activation Left Anterior Occipital Sulcus | 0.151 | 0.054 | 0.005 | 0.001 | 0.021 |
| 2-Back Activation Left Middle Frontal Sulcus | -0.127 | 0.046 | 0.005 | 0.001 | -0.006 |
| 2-Back Activation Left Lateral Orbital Sulcus | -0.086 | 0.032 | 0.006 | 0.001 | -0.002 |
| 2-Back Activation Right Middle-Anterior Cingulate | -0.170 | 0.062 | 0.006 | 0.001 | -0.023 |
| 2-Back Activation Right Horizontal Ramus of Anterior Lateral Sulcus | 0.121 | 0.045 | 0.007 | 0.001 | 0.031 |
| 2-Back Activation Left Superior Precentral Sulcus | -0.162 | 0.060 | 0.007 | 0.001 | -0.001 |
| 2-Back Activation Right Lateral Aspect of Superior Temporal Gyrus | 0.161 | 0.066 | 0.014 | 0.001 | 0.011 |
| 2-Back Activation Left Marginal branch of the cingulate sulcus | 0.147 | 0.064 | 0.021 | 0.001 | 0.01 |
| 2-Back Activation Left Medial Orbital Sulcus | 0.041 | 0.018 | 0.023 | 0.001 | 0.013 |
